# Effects and Acceptability of a 1-Week Home-Based Virtual Reality Training for Supporting the Management of Stress and Anxiety: Randomized Pilot Trial

**DOI:** 10.2196/50326

**Published:** 2025-03-06

**Authors:** Federica Pallavicini, Eleonora Orena, Lisa Arnoldi, Federica Achille, Stefano Stefanini, Maddalena Cassa, Alessandro Pepe, Guido Veronese, Luca Bernardelli, Francesca Sforza, Sara Fascendini, Carlo Alberto Defanti, Marco Gemma, Massimo Clerici, Giuseppe Riva, Fabrizia Mantovani

**Affiliations:** 1 Department of Human Sciences for Education “Riccardo Massa” University of Milano-Bicocca Milan Italy; 2 IRCCS Neurological Institute Carlo Besta Milan Italy; 3 European Biomedical Research Foundation Gazzaniga Italy; 4 Become-Hub Milan Italy; 5 Department of Medicine and Surgery University of Milano-Bicocca Milan Italy; 6 Humane Technology Lab Department of Psychology Catholic University of the Sacred Heart Milan Italy; 7 Applied Technology for Neuro-Psychology Lab IRCCS Istituto Auxologico Italiano Milan Italy

**Keywords:** virtual reality, relaxation, anxiety, depression, emotions, health care professionals, health care workers, hospital, randomized clinicial trial, hospitals

## Abstract

**Background:**

Virtual reality (VR) is helpful for the management of stress and anxiety. However, current interventions have limitations related to location (ie, therapist’s office or hospitals) and content (ie, virtual experiences only for relaxation).

**Objective:**

This randomized pilot trial aims to investigate the efficacy and acceptability of a brief remote VR-based training for supporting stress and anxiety management in a sample of Italian health care workers.

**Methods:**

A total of 29 doctors and nurses (n=21; 72% female; mean age 35.6, SD 10.3 years) were recruited and randomized to a VR intervention group or a control group in a passive control condition. Participants assigned to the VR intervention group received remote VR-based training consisting of 3 sessions at home delivered in 1 week using the VR psychoeducational experience “MIND-VR” and the 360° relaxing video “The Secret Garden.” The primary outcome measures were stress, anxiety, depression, and the knowledge of stress and anxiety assessed at baseline and posttreatment. We also evaluated the immediate effect of the remote VR-based training sessions on the perceived state of anxiety and negative and positive emotions. The secondary outcome measure was the usability at home of the VR system and content.

**Results:**

The VR intervention significantly reduced stress levels as assessed by the Perceived Stress Scale (6.46, 95% CI 2.77 to 10.5; *P*=.046) and increased the knowledge of stress and anxiety, as evaluated by the ad hoc questionnaire adopted (–2.09, 95% CI –3.86 to –0.529; *P*=.046). However, the home-based VR training did not yield similar reductions in stress, anxiety, and depression levels as assessed by the Depression, Anxiety, and Stress Scale-21 items or in trait anxiety as evaluated through the State-Trait Anxiety Inventory Form Y-1. After the home training sessions with VR, there was a significant decrease in anxiety, anger, and sadness and an increase in happiness levels. Analyses of the questionnaires on usability indicated that the health care workers found using the VR system at home easy and without adverse effects related to cybersickness. Of 33 participants, 29 (88%) adhered to the protocol.

**Conclusions:**

The results of this randomized pilot study suggest that a week-long home VR intervention, created with content created specifically for this purpose and available free of charge, can help individuals manage stress and anxiety, encouraging further research investigating the potential of remote VR interventions to support mental health.

**Trial Registration:**

ClinicalTrials.gov NCT04611399; https://tinyurl.com/scxunprd

## Introduction

### Background

Virtual reality (VR) is defined as a set of technologies, including a head-mounted display (HMD), computer, and mobile devices, that allow users to interact with a 3D environment in real time [1]. This technology has been rapidly advancing in recent years, with various applications ranging from gaming and entertainment to education and health care [2-5]. In the last decade, VR has become a cutting-edge technology that could provide enhanced value for health care, representing a helpful and practical instrument for psychological support [6-8].

Several studies, reviews, and meta-analyses have shown that VR is useful for decreasing stress, anxiety, and negative emotions, generating positive emotional states [9-11].

Relaxing VR content, especially those created ad hoc by researchers and clinicians to mimic nature [9,12] and immersive video games [11,13], have been shown to help individuals learn and practice relaxation techniques, such as breath exercise [14], progressive muscle relaxation [15], biofeedback [16,17], and mindfulness [18,19]. Additionally, VR can also be an effective tool for psychoeducation, an intervention that provides individuals with information about their illness and teaches them coping skills [20-23].

The efficacy of VR to support the management of stress and anxiety has been shown since the 2000s to be useful in different populations of people, both healthy and enduring various mental and physical disorders [5,24]. This fact appears important because stress and anxiety are common mental health conditions that have become increasingly prevalent, especially since the COVID-19 pandemic [25,26], with a prevalence of 29.6% and 31.9% [26].

One group that is particularly affected by stress and anxiety is health care workers. Some of their most common causes of stress and anxiety include long working hours and constant exposure to illness and death [27,28]. The COVID-19 pandemic has also added an extra layer of stress and anxiety among health care workers [29,30]. The high prevalence of stress and anxiety among health care workers is a significant concern for public health. It affects the well-being of health care workers and has implications for patient care and safety [31,32].

Interestingly, recent studies showed the usefulness of VR-based interventions for diminishing stress and anxiety among health care workers. For example, watching 360° videos of calming natural environments was effective for relaxation in a sample of ICU [33]. Furthermore, a 3-minute immersive video of a nature scene was found to lower stress levels among frontline health care workers in COVID-19 treatment units [34].

Although the use of VR for supporting the management of stress and anxiety has an increasingly solid scientific basis, there are two limitations still limiting its adoption and usefulness, both among health care professionals and more broadly. First, in past studies, experienced clinicians only administered VR interventions in their offices or the hospital. However, recently the introduction of standalone and mobile VR systems, due to their high ease of use and low cost, has made this technology feasible for daily use at home [35]. Therefore, home VR training represents a promising new intervention for remote psychological support [36-38], including relaxation training [39,40]. However, little is currently known about the usefulness and acceptability of VR-based home programs.

Second, in previous studies that have used VR for stress and anxiety management, the proposed intervention involved using VR only for relaxation [33,34]. None of these also incorporated a part of psychoeducation. However, as suggested in the literature, psychoeducation is important for stress and anxiety management, as it could increase treatment adherence and reduce self-esteem [41]. By providing individuals with an understanding of the nature of anxiety and stress, along with practical coping strategies, psychoeducation empowers them to manage these challenges effectively [42,43]. It also plays a preventive role by raising awareness and reducing the stigma surrounding these issues, thus potentially averting more severe mental health problems [44,45].

### Aims of This Study

Within the context described above, the main aim of the present randomized pilot trial was to evaluate the effectiveness and acceptability of a home-based VR intervention for managing stress and anxiety in a sample of Italian health care workers.

The primary objective of this randomized pilot trial focuses on the usefulness of the proposed remote VR-based intervention for decreasing stress and anxiety and enhancing the knowledge about these conditions compared to a passive group as the control condition. The second objective was to evaluate the immediate effect on stress, anxiety, and negative and positive emotions of at-home VR training sessions. Finally, the third objective was to assess the usability at home of the VR system and content.

## Methods

### Study Design

This pilot trial followed a 2-arm, parallel-group, randomized controlled trial model with between-subjects repeated measures (pre and post) with VR intervention as the experimental condition and a passive group as the control condition. The introduction and methods sections are based on the published protocol [46].

### Ethical Considerations

The research was approved by the Ethics Committee of the University of Milan-Bicocca (ie, approval 0061757/20 on September 25, 2020) and of the Foundation IRCCS Carlo Besta Neurological Institute Foundation (ie, approval number 75 on September 16, 2020). This study followed the ethical standards outlined in the Declaration of Helsinki (1964) and its subsequent revisions. Before data collection, the plan for this randomized pilot trial’s design, data collection, and analysis was registered in the ClinicalTrials.gov database (NCT04611399) on February 11, 2020.

Before taking part in this study, participants filled out an informed consent form. The form explained the procedures, risks, and benefits of participating in the research, ensuring confidentiality of personal data, and adherence to ethical principles. The informed consent also stated that participation was voluntary, and the participants could withdraw at any time. All data gathered during this study were stored anonymously in a web central database repository. Every individual was assigned a unique participant number consisting of name and surname first letters and a consecutive number. This unique code identified all participant-specific data (eg, epidemiological and clinical study data). Data access and storage followed the data security, including password-protected access to all computers and folders. The participants received no incentives or rewards to participate.

The trial was conducted at 2 medical sites in Italy: the Foundation IRCCS Carlo Besta Neurological Institute Foundation and the Fondazione Europea Ricerca Biomedica. In each medical site, the participants were recruited from hospital units that treated patients with COVID-19 (ie, the emergency department, surgical units, and critical care units). Health care workers were informed about the possibility of participating in this study with oral communication and a formal email from the institutional study referent. The participants had to confirm their intention to participate in this study by responding to the email while the contact person set up an appointment for the screening interview, as described below.

### Participants

The participants’ eligibility was verified through a screening interview with the research psychologist, who explained the training’s aims and methods and conducted an assessment to identify the participants who could enter this study. This study’s inclusion criteria were the following: (1) being currently employed as a health care worker, (2) maximum age of 65 years, (3) absence of medical disorders (heart disease or blood pressure, neurological disorders, or epilepsy), (4) absence of pharmacotherapy that could interfere with the measured data (psychoactive drugs, antihypertensive, or antidepressants), and (5) no significant visual impairment (all with normal visual acuity or corrected to normal).

A total of 43 individuals were assessed for eligibility to participate in this study: 10 (23%) participants did not consent to participate, and 4 (9%) did not complete the posttreatment data and were considered dropouts. For the analyses of the long-term impact of the intervention, we used a complete case analysis. By focusing on the participants with complete data, we aimed to minimize potential bias while maximizing the use of available data.

Out of 33 participants, 29 (88%) adhered to the protocol: 21 (72%) females and 8 (28%) males; mean age of 35.6 (SD 10.3) years; mean years of professional seniority of 10.1 (SD 10.2) years. Further, 22 (76%) participants were from the Foundation IRCCS Carlo Besta Neurological Institute Foundation and 7 (24%) from the Fondazione Europea Ricerca Biomedica. Furthermore, 18 (62%) individuals were doctors and 11 (38%) were nurses or social health workers.

### Sample Size Estimation

A formal sample size calculation was not feasible due to the absence of data that could serve as the foundation for the calculation. However, for pilot studies with an anticipated moderate standardized effect size, enrolling a minimum of 15 individuals per group is recommended to achieve a power of 90% for the subsequent main studies [47].

### Randomization and Blinding

Before this study commenced, the participants were randomly assigned to the experimental or control group in a 1:1 manner. This study’s coordinator created the randomized list with a block randomization procedure using a true random number generator [48]. During the screening interview, eligible individuals who met the inclusion criteria described above received written information about the procedure and were asked to sign the consent form to participate in this study. Only qualified individuals who provided informed consent were randomly assigned to the experimental or control group. The participants received information regarding the allocation result during the screening interview.

### Interventions

#### VR Intervention

We describe the protocol in detail in the following section.

Session 1 was the intake session. Once the participants signed the informed consent and completed the online baseline questionnaire (ie, demographic questions, ad hoc questionnaire on technological solutions and VR, Perceived Stress Scale [PSS-10], State-Trait Anxiety Inventory Form Y-2 [STAI-Y2], Depression, Anxiety and Stress Scale-21 items [DASS-21], and an ad hoc questionnaire on knowledge of stress and anxiety), a research psychologist provided them with a detailed explanation of the aims and methodology of the 1-week home-based VR program. For the intervention in the VR group, we used the Oculus Quest 2 (Facebook Technologies LLC), a consumer-grade standalone VR system that consists of an HMD and 2 controllers. The Oculus Quest 2 offers a single LCD panel and 1832 × 1920-pixel resolution per eye, with a 90 Hz refresh rate. The participants received a brief 15-minute training on using the VR system and were then given the Oculus Quest 2 to take home.

**Figure 1:**
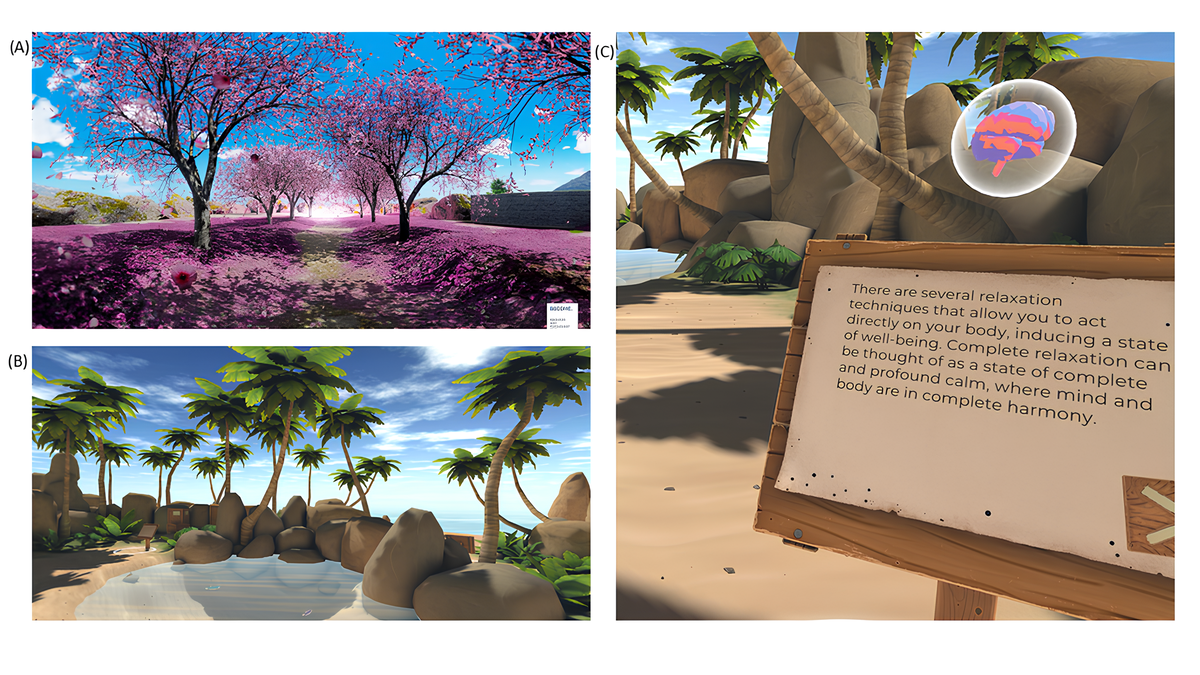
Screenshots of (A) “The Secret Garden” and (B, C) “MIND-VR.” VR: virtual reality.

Training sessions (sessions 2, 3, and 4) consisted of 3 home sessions of approximately 30 minutes each, conducted in 1 week with a distance of 2 days between 1 session and another. The participants tried for about 15 minutes “MIND-VR,” a VR-based psychoeducational experience on stress and anxiety created by a team from the University of Milano-Bicocca in collaboration with AnotheReality [21]. The user explored one of three areas within a virtual island in each session. Each focused on different aspects related to stress and anxiety (ie, definitions, causes, symptoms, and main treatments). Subsequently, the participants used the VR relaxation content “The Secret Garden,” a 10-minute computer graphic 360° video developed by Riva et al [39,40] for relaxation training freely available on the website (Figure 1) [49]. At the beginning and the end of each session, the participants were asked to complete online the visual analog scale for anxiety (VAS-A) and the State-Trait Anxiety Inventory Form Y-1 (STAI-Y1).

Session 5 was the posttraining session. At the end of the training, the research psychologist met the participants for the postintervention assessment. On this occasion, the participants returned the Oculus Quest 2. The participants were asked to complete the online questionnaires within 1 week after completion of the intervention (ie, PSS-10, STAI-Y2, DASS-21, ad hoc questionnaire on knowledge of stress and anxiety, System Usability Score [SUS], Subjective Difficulty Measure [SDM], Net Promoter Score [NPS], and ad hoc questionnaire on difficulties and adverse effects).

#### Control Group

This group will undergo baseline and postintervention assessments without undergoing any training during the 1-week intervention period.

### Measures

#### Primary Outcome Measure

At baseline and postintervention, the participants in the VR and the control group completed the following questionnaires:

- PSS-10 [50]: the PSS is a 10-item self-reported measure to assess the current stress level.
- STAI-Y2 [51]: a validated and widely used measure of trait anxiety.
- DASS-21 [52]: a set of 3 self-report scales designed to measure depression, anxiety, and stress symptoms.
- Ad hoc questionnaire on the knowledge of stress and anxiety: the participants completed 4 factual questions in a multiple-choice format (eg, “Which is the first phase of the General Adaptation Syndrome?”) and 2 conceptual questions in a short-answer format (eg, “What are the three main categories of stress symptoms?”). The questions were based on the methodology used in a previous study [53]. The 4 factual questions are given a score of 0 if the answer is incorrect and 1 if correct, while the 2 conceptual questions are given a score of 0 to 3 depending on the number of correct items entered. The maximum total score is 10.

#### Secondary Outcome Measure

Postintervention, the participants in the experimental group filled out the following questionnaires that served as the secondary outcome measures:

- SUS [54]: a measure of usability aspects (ie, efficiency, clarity, or reliability). The participant’s scores for each question are converted into a new number, added together, and then multiplied by 2.5 to convert the original scores from 0-40 to 0-100. Based on the research, an SUS score above 68 would be considered above average, while any score below 68 is below average (eg, [55,56]).
- SDM: a horizontal line 100 mm long, anchored by word descriptors at each end, from “not at all” to “very much.” The users mark on the line the perceived difficulty in using the VR system, “MIND-VR,” and “The Secret Garden” on a scale from 0 (“not at all”) to 10 (“very much”). The lower the score, the lower the perceived difficulty.
- NPS [57,58]: it evaluates user satisfaction with a product, in this case: the home-based VR intervention in general, “MIND-VR,” and “The Secret Garden.” It requires the participants to indicate on a scale from 0 (“not at all”) to 10 (“very much”) how much they would recommend the products to a family member or friend. The scoring procedure involves categorizing respondents into 3 groups based on their ratings: promoters (scores of 9 or 10), passives (scores of 7 or 8), and detractors (scores of 0 to 6). Then, the percentages of respondents in each category are calculated by dividing the number of respondents in each group by the total number of respondents and multiplying by 100. Finally, the NPS is determined by subtracting the percentage of detractors from the percentage of promoters using the formula: NPS = % promoters – % detractors. The resulting NPS can range from –100 to +100. A positive score indicates that there are more promoters than detractors, while a negative score suggests the opposite. A higher NPS generally indicates higher customer satisfaction and loyalty [57,58].
- Ad hoc questionnaire on difficulties and adverse effects: individuals were asked to rate on a 7-point Likert scale (1=“not at all” and 7=“very much”): If they had difficulty following the experimental protocol; if they had nausea, headache, dizziness, and eyestrain while using the VR system in general; if they had nausea, headache, dizziness, and eyestrain while using “MIND-VR”; if they had nausea, headache, dizziness, and eyestrain experienced while using “The Secret Garden.” The Cronbach α coefficient of the questionnaire was 0.96.

#### Other Measures

At baseline, the participants in the VR and the control group completed the following: (1) Demographic: genre, age, years of education, profession, hospital, work department, and years of professional seniority. (2) Ad hoc questionnaire on the use of VR: the individuals were asked to indicate whether they had ever tried VR before and their level of knowledge of this technology. (3) Besides, to measure changes in the affective states of the individuals during the intervention, the participants of the experimental group were asked to fill in before and after each of the training sessions the following self-report questionnaires: (a) STAI-Y1 [51], used to assess state anxiety (ie, a temporary emotional condition characterized by apprehension, tension, and fear about a particular situation or activity). (b) VAS-A [59], a horizontal line on a scale from 0 to 100, anchored by word descriptors at each end (“no anxiety” and “very severe anxiety”). The individuals mark on the line the point that they feel represents their perception of their current level of state anxiety. (c) Visual analog scale for emotions (VAS-E) [59]—the participants indicated on a scale from 0 to 100 their current experienced level of 6 primary emotions: anger, happiness, disgust, fear, sadness, and surprise. Several studies have confirmed its reliability and validity (eg, [60]).

### Statistical Analyses

Data analyses were carried out through IBM SPSS Statistics (version 25.0 software package for Windows; SPSS Inc) software. ANOVA was used to evaluate baseline characteristics of the 2 groups involved in this study and the overall significance of improvement across primary outcome measures. Categorical variables were compared using Fisher or chi-square tests, and continuous variables using *t* tests (2-tailed) or Mann-Whitney tests, as appropriate.

We used repeated ANOVA for the primary end point outcome. Time was treated as a categorical variable, and the models included group, time, and group-by-time interaction as fixed effects. The conclusions about the usefulness of the VR intervention were based on between-session comparisons of change in PSS-10, STAY-Y2, and DASS-21, and in a questionnaire on the knowledge of stress and anxiety from baseline to posttreatment. Given the relatively small sample, bootstrapping (number of samples: 1000) was used to increase the robustness of statistical analyses. This approach allowed us to estimate the variability of results more accurately and to obtain more precise estimates of the parameters of interest.

A 2×3 repeated measures ANOVA was used to analyze the within-session change in the situational state anxiety (STAI-Y1 and VAS-A) and negative and positive emotions (VAS-E) scores before and after each home-based VR training session. Tests of statistical significance and CIs were 2-sided. A *P*<.05 was considered statistically significant. Descriptive methods were used to report the usability at homes of the VR system and content for the secondary outcome intervention (ie, SUS, SDM, NPS, and ad hoc questionnaire on difficulties and adverse effects).

## Results

### Descriptive Statistics and Baseline Characteristics

**Figure 2:**
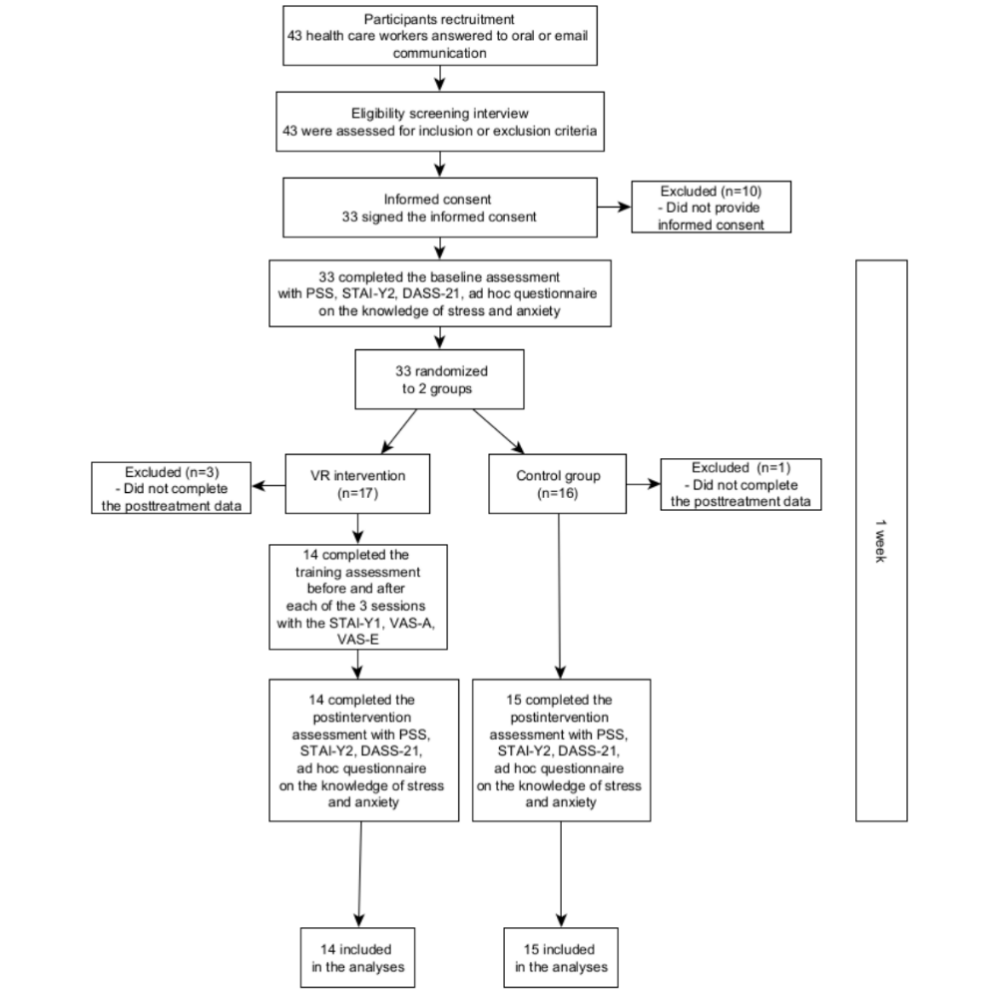
Trial flow chart. DASS-21: Depression, Anxiety, and Stress Scale-21 items; PSS: Perceived Stress Scale; STAI-Y1: State-Trait Anxiety Inventory Form Y-1; STAI-Y2: State-Trait Anxiety Inventory Form Y-2; VAS-A: visual analog scale for anxiety; VAS-E: visual analog scale for emotions.

The trial flow chart is summarized in Figure 2. The presence of spurious associations among demographic variables and experimental groups was evaluated by using a chi-square test of independence and *t* test analyses for independent samples (Table 1). The results highlighted that no statistically significant associations were found among considered variables in the 2 groups.

Concerning other the participants’ characteristics, baseline measures did not report statistically significant differences between the 2 groups, meaning that variable scores were homogeneous (Table 2).

**Table 1:** Chi-square test of independence on gender and previous experience with VR in the 2 experimental groups.^a^

Variables		VR group (n=15)	Control group (n=14)	Chi-square (*df*)		*P* value	
**Gender**					0.013 (1)		.91
	Female	11	10				
	Male	4	4				
**Previous experience with VR**					0.024 (1)		.87
	Yes	6	6				
	No	9	8				

^a^VR: virtual reality.

**Table 2:** Mean comparison of demographic and primary outcome measures assessed at baseline in the 2 experimental conditions (VR group: n=15; control group: n=14).^a^

Variables			Mean (SD)		*t* test (*df*)		*P* value
**Age (years)**					–1.3 (27)		.2
	VR	33.2 (10)					
	Control	38.1 (10.3)					
**Years of professional seniority**					–1.27 (27)		.21
	VR	7.8 (9.4)					
	Control	12.5 (10.7)					
**Knowledge of VR**					–0.087 (27)		.43
	VR	1.53 (0.743)					
	Control	1.86 (1.35)					
**PSS-10^b^**					0.005 (27)		.99
	VR	20.8 (5.7)					
	Control	20.8 (5.1)					
**STAI-Y2^c^**					0.121 (27)		.90
	VR	46.8 (12.6)					
	Control	46.2 (13.2)					
**DASS-21^d^ stress**					0.614 (27)		.55
	VR	28.1 (10.3)					
	Control	26 (8.2)					
**DASS-21 anxiety**					0.263 (27)		.79
	VR	20.1 (5.5)					
	Control	19.5 (5.9)					
**DASS-21 depression**					1.196 (27)		.24
	VR	23.1 (4.5)					
	Control	20.5 (6.5)					
**Knowledge of stress and anxiety**					–0.475 (27)		.64
	VR	4.8 (2.6)					
	Control	5.2 (2.01)					

^a^VR: virtual reality.

^b^PSS-10: Perceived Stress Scale.

^c^STAI-Y2: State-Trait Anxiety Inventory Form Y-2.

^d^DASS-21: Depression, Anxiety, and Stress Scale-21 items.

### Impact of the VR Intervention on Primary Outcomes

To investigate whether a home-based VR intervention can support health care workers in managing stress and anxiety, we compared the effects of the VR intervention and control condition on the primary outcome measures. The average change in primary outcome measures for the VR and control conditions from baseline to postintervention is depicted in Table 3.

**Figure 3:**
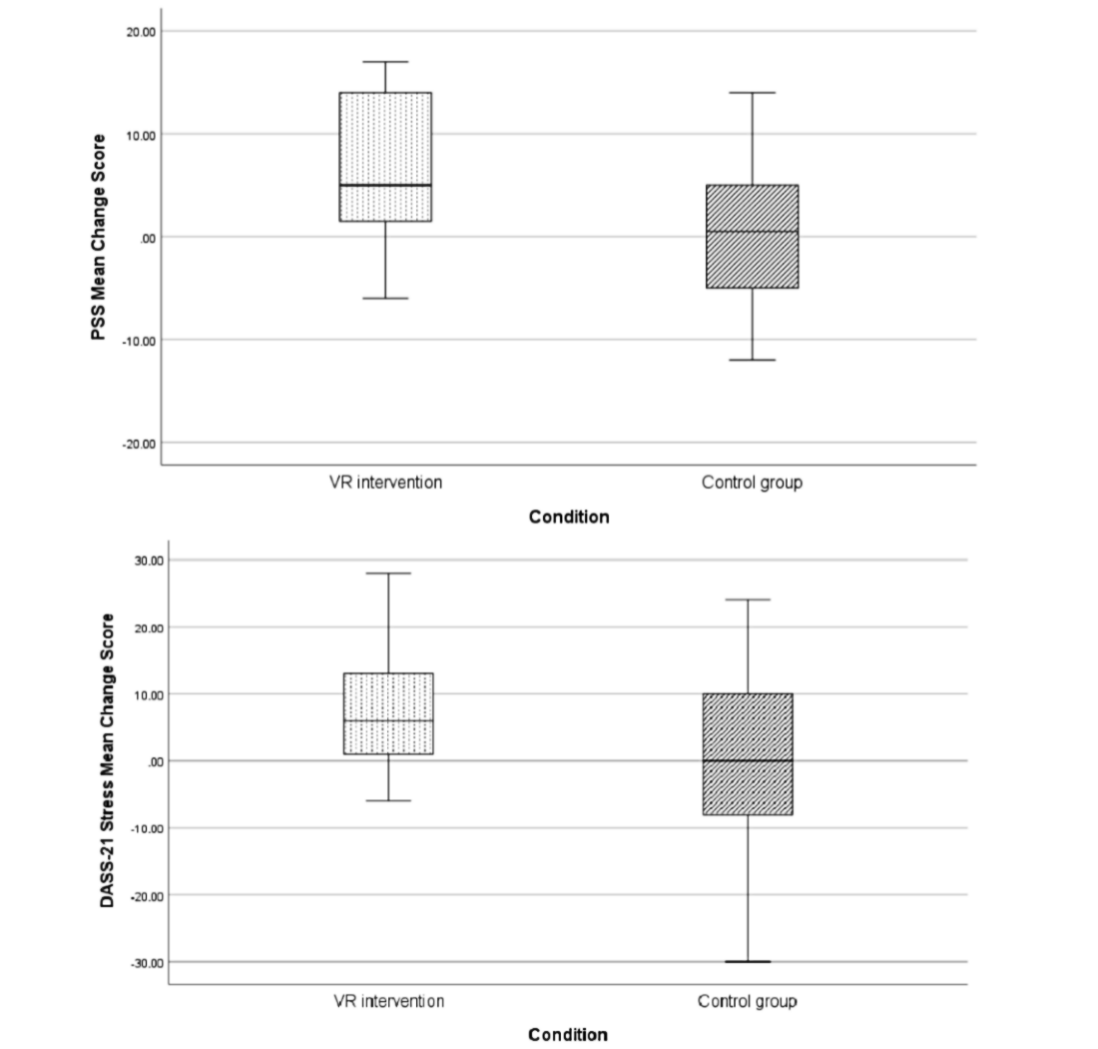
Change in the stress level as assessed with PSS-10 and DASS-21 from baseline measurement to posttreatment (1 week) for both conditions. Positive values indicate a decrease in stress levels. Error bars are 95% CIs. DASS-21: Depression, Anxiety, and Stress Scale-21 items; PSS-10: Perceived Stress Scale; VR: virtual reality.

The VR intervention led to a decrease in PSS-10 scores (mean 6.46, SD 7.41; 95% CI 2.77 to 10.5), which was significantly larger than the control condition (mean 0.785, SD 7.43; 95% CI –2.93 to 4.66; t_27_=2.06; *P*=.046). The effect size calculated as Cohen *d* of the reported change in the VR intervention compared to the control condition was high, *d*=0.765. Figure 3 shows a significant difference between the VR and control conditions in their average change in perceived stress level from baseline to after the intervention as measured by the PSS-10.

**Figure 4:**
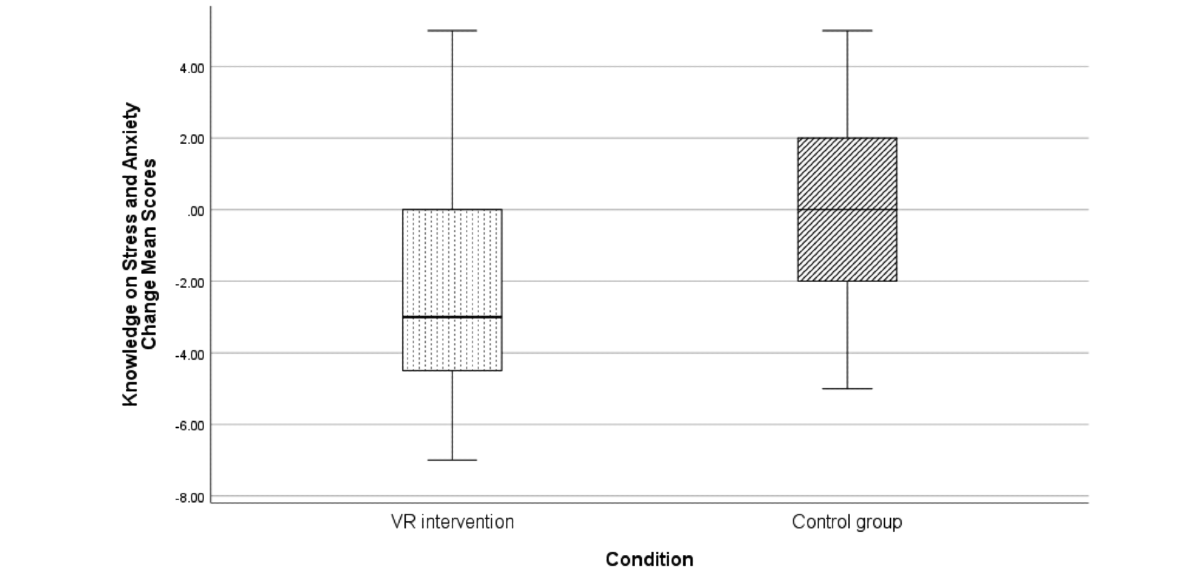
Change in the knowledge of stress and anxiety as measured by the ad hoc questionnaire from baseline measurement to posttreatment (1 week) for both conditions. Negative values indicate an increase in the knowledge of stress and anxiety. Error bars are 95% CIs. VR: virtual reality.

However, results did not reveal similar reductions in stress, anxiety, and depression levels as measured by the DASS-21 or in trait anxiety as assessed by the STAI-Y1 (Table 3). Furthermore, results showed that the VR intervention led to an increase in the knowledge of stress and anxiety, as assessed by the ad hoc questionnaire adopted (mean –2.09, SD 3.28; 95% CI –3.86 to –0.529) significantly greater than in the control condition (mean 0.71, SD 2.67; 95% CI –1.33 to 1.38; t_27_=–2.09; *P*=.046; Figure 4). The effect size calculated as Cohen *d* of the reported change in the VR intervention compared to the control condition was high (*d*=–0.778).

**Table 3:** Comparison of the effect of the home-based VR^a^ intervention and control condition on scores on PSS-10^b^, STAI-Y2^c^, DASS-21^d^, and ad hoc questionnaire on the use of VR. All variables are calculated as change scores from the baseline to the second measurement.

Variables	Control (n=14), mean (SD)	VR intervention (n=15), mean (SD)	*t* test	*P* value	Bootstrap *P* value	Bootstrap 95% CI
PSS-10	0.785 (7.43)	6.46 (7.41)	2.06	.049	.046	0.29 to 10.8
STAI-Y2 (trait)	3.38 (15.1)	2.06 (15.9)	–0.211	.83	.83	–12 to 10.8
DASS-21 stress	–1.85 (16.1)	8.53 (10.6)	2.06	.049	.053	1.2 to 20.6
DASS-21 anxiety	–2 (10.6)	4 (5.34)	1.93	.06	.08	0.87 to 12.3
DASS-21 depression	–2.57 (13.39)	5.06 (5.11)	2.05	.05	.06	0.14 to 14.7
Knowledge of stress and anxiety	0.71 (2.67)	–2.26 (3.28)	–2.09	.046	.046	–4.4 to –1.2

^a^VR: virtual reality.

^b^PSS-10: Perceived Stress Scale.

^c^STAI-Y2: State-Trait Anxiety Inventory Form Y-2.

^d^DASS-21: Depression, Anxiety, and Stress Scale-21 items.

### Immediate Effects of the VR Intervention on Secondary Outcomes

A 2×3 repeated measures ANOVA revealed a significant effect of time in the STAI-Y1 (*F*_1,14_=5.8; *P*=.03; η2=0.293) and VAS-A (*F*_1,14_=5.33; *P*=.03; η2=0.276), indicating that the VR training sessions were able to decrease state anxiety. Besides, results on the VAS-E showed a statistically significant difference (*P*=.05) before and after the use of “MIND-VR” in the scores at VAS happiness (*F*_1,14_=4.56; *P=*.05; VAS-A; η2=0.246), VAS anger (*F*_1,14_=7.8; *P*=.10; VAS-A; η2=0.358), and VAS sadness (*F*_1,14_=8.53; *P*=.01; η2=0.279), indicating that the training sessions using VR were useful for enhancing happiness and decreasing sadness and anger (Table 4).

**Table 4:** Mean changes in state anxiety as assessed with STAI-Y1^a^ and VAS-A^b^, and positive and negative emotions as measured through VAS-E^c^ in the 3 training sessions of the home-based VR^d^ intervention.

Variables			Pre mean (SD)		Post mean (SD)		Effects		*F* test (*df*)		*P* value		η2
**STAI-Y1**													
	Session 1	41.4 (14.3)		33.4 (8.7)		Time		5.8 (1,14)		.03		0.293	
	Session 2	42.4 (13.4)		37.7 (13.9)		Session		0.45 (1,14)		.65		0.065	
	Session 3	40.4 (12)		36.8 (11.5)		Time × session		0.465 (1,14)		.64		0.067	
**VAS-A**													
	Session 1	36.7 (23.5)		20.7 (20.8)		Time		5.33 (1,14)		.04		0.276	
	Session 2	33.3 (20.9)		24 (23.8)		Session		0.069 (1,14)		.93		0.011	
	Session 3	32 (22.7)		28 (19.3)		Time × session		1.15 (1,14)		.35		0.151	
**VAS-DIS^e^**													
	Session 1	9.3 (19.8)		5.3 (20.7)		Time		0.723 (1,14)		.41		0.049	
	Session 2	12.7 (25.7)		9.3 (24.6)		Session		3.08 (1,14)		.08		0.322	
	Session 3	7.3 (15.7)		5.3 (15.5)		Time × session		0.072 (1,14)		.93		0.011	
**VAS-HP^f^**													
	Session 1	56.7 (23.1)		62.6 (11.7)		Time		4.56 (1,14)		.05		0.246	
	Session 2	51.3 (24.4)		62 (15.2)		Session		0.171 (1,14)		.84		0.026	
	Session 3	50.7 (24)		64 (15.5)		Time × session		0.638 (1,14)		.54		0.089	
**VAS-AG^g^**													
	Session 1	20.7 (27.9)		6.7 (16.3)		Time		7.8 (1,14)		.01		0.358	
	Session 2	16 (25.5)		10 (19.6)		Session		0.041 (1,14)		.96		0.006	
	Session 3	16.7 (22.8)		12 (17.4)		Time × session		0.528 (1,14)		.60		0.075	
**VAS-FE** ^h^													
	Session 1	31.3 (30.6)		14 (22.6)		Time		2.83 (1,14)		.11		0.168	
	Session 2	15.3 (18.8)		12.7 (23.4)		Session		1.4 (1,14)		.28		0.177	
	Session 3	16 (23.5)		12.7 (23.1)		Time × session		0.962 (1,14)		.41		0.129	
**VAS-SP^i^**													
	Session 1	18 (20.7)		16 (20.9)		Time		0.003 (1,14)		.95		0.006	
	Session 2	8.7 (14)		11.3 (17.6)		Session		2.21 (1,14)		.15		0.254	
	Session 3	10.7 (18.7)		10.7 (18.7)		Time × session		0.958 (1,14)		.41		0.128	
**VAS-SD^j^**													
	Session 1	36 (37.5)		19.3 (27.6)		Time		8.53 (1,14)		.01		.379	
	Session 2	28.7 (26.7)		21.3 (20.9)		Session		0.773 (1,14)		.48		0.106	
	Session 3	26 (28.2)		15.3 (20.9)		Time × session		0.383 (1,14)		.69		0.056	

^a^STAI-Y2: State-Trait Anxiety Inventory Form Y-2.

^b^VAS-A: visual analog scale for anxiety.

^c^VAS-E: visual analog scale for emotions.

^d^VR: virtual reality.

^e^VAS-DIS: visual analog scale for disgust.

^f^VAS-HP: visual analog scale for happiness.

^g^VAS-AG: visual analog scale for anger.

^h^VAS-FE: visual analog scale for fear.

^i^VAS-SP: visual analog scale for surprise.

^j^VAS-SD: visual analog scale for sadness.

### Secondary Outcomes: Usability at Home of the VR System and Content

Regarding the usability at home of the VR system, the mean scores at the SUS were greater than 68 (mean 78, SD 12.8; 95% CI 70-86.5), indicating a high usability. Regarding SDM, the scores were low both in general (mean 2.5, SD 1.1; 95% CI 1.93-3.07) and concerning “MIND-VR” (mean 2.3, SD 1.03; 95% CI 1.7-2.7) and “The Secret Garden” (mean 2.3, SD 1.1; 95% CI 1.7-2.8), showing that the VR system and content were perceived as easy-to-use. In addition, results showed low scores in the adverse effects of VR use related to cybersickness symptoms, both overall (mean 2.9, SD 1.7; 95% CI 2.13-3.8) and concerning “MIND-VR” (mean 2.4, SD 1.4; 95% CI 1.8-3.2) and “The Secret Garden” (mean 2.4, SD 1.4; 95% CI 1.73-3.13), indicating that the use of VR had no effects related to cybersickness. As for the NPS, however, it was –13 concerning the program as a whole, –33 for “MIND-VR,” and –6 for “The Secret Garden,” suggesting varying levels of satisfaction among the participants.

## Discussion

### Principal Findings

This pilot trial delivered clinical and feasibility data on a home-based VR intervention for supporting stress and anxiety management in a sample of health care workers, one of the categories most affected by these conditions and their adverse effects.

Starting with the primary outcome measures, the results of this randomized pilot trial showed that, compared to the passive control condition, our 1-week remote VR-based intervention significantly reduced the level of perceived stress as assessed by the PSS-10. This reduction is underscored by the notable effect size of *d*=0.765, suggesting a meaningful impact of the intervention. However, the home-based VR training did not yield similar reductions in stress, anxiety, and depression levels as assessed by the DASS-21 or in trait anxiety as evaluated through the STAI-Y1.

The discrepancy in results, wherein a reduction was noticed in perceived stress levels as evaluated by the PSS-10 but not reflected in the outcomes from the DASS-21, suggests potential differences in the sensitivity or specificity of these measures in capturing changes following the VR intervention. It could also imply that the VR intervention primarily targeted aspects of stress that are more closely aligned with the constructs measured by the PSS-10, highlighting the importance of selecting appropriate outcome measures tailored to the specific objectives of the intervention.

Furthermore, the fact that the results showed no differences in anxiety and depression levels as assessed by the DASS-21 or in trait anxiety as evaluated through the STAI-Y1 could be explained by the short duration of the training. In support of this hypothesis, also in the study by Riva et al [40] on the use of VR to assist individuals in coping with the psychological burden related to the COVID-19 pandemic, the use at home for one week of a 360° video for relaxation (ie, “The Secret Garden,” the same one used in our study), used through a low-cost cardboard HMD, had brought improvements in stress levels, but not for the perceived anxiety levels (as measured with the DASS-21) [40]. In contrast, a reduction in trait anxiety was observed after a program with VR lasting 5 weeks, consisting of 8 sessions based on biofeedback techniques, and delivered at the therapist’s office [15]. Future studies must deeply investigate the optimal duration of remote training for VR-based stress and anxiety management to maximize its effectiveness.

Regarding the level of anxiety and stress knowledge, the results indicated a significant change between different time points (baseline vs end of intervention assessment) after the home VR intervention. Specifically, compared with the passive control condition, the VR intervention significantly increased knowledge of anxiety and stress, with an effect size high (*d*=–0.778).

This result, in line with previous literature [21,61,62], stresses the potential of VR as a valuable and innovative tool for promoting scientific and medical knowledge on stress and anxiety and other mental disorders such as depression [22,23]. Through immersion in the VR environment, individuals may feel secure and free from judgment, leading to a greater understanding of their mental health condition [22]. Greater awareness of symptoms can have significant implications for clinical outcomes, including treatment-seeking, adherence, and recovery [63,64], decreasing hospitalization rates, and saving long-term physician consultation costs. More studies are needed to expand the limited literature on VR-based psychoeducation. For example, it would be interesting to test whether inserting engaging elements and guiding cues before the VR psychoeducational experience can increase effectiveness in learning. Equally important is testing the total duration of the psychoeducation program in VR, trying to propose interventions of longer duration than the one proposed in this study (ie, 1-week).

Results for the secondary outcomes indicated that the home VR training sessions immediately decreased perceived stress and anxiety levels. Specifically, the results of the analyses on STAI-Y1, VAS-A, and VAS-E showed that after the use of the psychoeducational (ie, “MIND-VR”) and relaxing (ie, “The Secret Garden”) VR experience, a significant increase in the intensity of positive emotions (ie, happiness), a significant decrease after VR home training sessions in the levels of anxiety, anger, and sadness, and an increase in the levels of happiness was experienced. This fact appears important as it points out how even using VR at home for short periods can help induce a state of relaxation in the individual, with immediate positive psychological effects. As underlined by the broaden-and-build model [65], experiencing positive emotions can enhance interaction with others or engagement in creative challenges. Besides, a positive emotional state positively impacts learning processes, promoting acquiring the information provided [66].

This study’s virtual content can be easily and affordably incorporated as supplementary treatment or ongoing support to improve the efficacy of established evidence-based interventions for health care workers. It could also be readily modified to help other groups dealing with stress and anxiety. “MIND-VR” and “The Secret Garden” are available for free in multiple languages, including English. As the use of free content could decrease the cost and accessibility of care support, it seems essential that future studies study the topic in detail, identifying other content potentially beneficial for both stress and anxiety management and other conditions, such as VR video games or naturalistic experiences.

Finally, regarding the usability at home of the VR system and content, analyses of questionnaires (ie, SUS, SDM, or ad hoc questionnaires on adverse effects) indicated that health care workers found the VR system easy to use and without adverse effects related to cybersickness symptoms (eg, nausea and vertigo). Moreover, both the VR psychoeducational experience (“MIND-VR”) and the relaxation content (“The Secret Garden”) were reported as easy to use and did not induce cybersickness–related effects. These findings offer preliminary data supporting the ease of use and lack of adverse effects of VR used in the home, even in individuals without high knowledge of this technology and of different genders and ages, as in our sample case. As the use of VR in home settings can increase participation, involvement, and compliance to follow psychological support programs, increasing treatment adherence and lowering self-stigma [67,68], future studies should deeply investigate this topic.

However, it is important to highlight that from the results of the NPS analysis, a percentage of the individuals were not satisfied with the training or content, suggesting an opportunity for improvement. To gain valuable insights into areas where changes need to be made, it will be important to carefully examine the participants’ feedback also from a qualitative perspective, such as through interviews and focus groups.

The results of this study should be interpreted based on several additional potential limitations. First, in this study, a third control arm (ie, treatment as usual) was not included due to the limitation of cost and feasibility. While it has been suggested that there may not be a significant difference in measured outcomes between active and passive control groups [69], future research needs to compare the effectiveness of the VR intervention with other methods. For example, future studies could explore the comparative effectiveness of VR interventions with placebo interventions and with traditional therapy approaches such as cognitive behavioral therapy or exposure therapy, while also investigating the synergistic effects of combining VR with mindfulness techniques for stress reduction and anxiety management.

Second, in this study, we used a full case analysis instead of following intention-to-treat principles. The analysis was conducted on the complete data (per protocol), believing that this approach was more appropriate to the main objective of this study, which was to evaluate the feasibility, acceptability, and potential preliminary impact of the intervention in a small group of participants. Pilot studies are not intended to provide definitive estimates of efficacy, but rather to test methodologies and gather useful information for planning future larger studies. In this context, the priority was not to obtain generalizable results but to explore the practical applicability of the protocol and identify potential critical issues. In addition, the small sample and missing data (n=4, 12%, dropout) make the application of imputation techniques delicate, which could have introduced significant bias. Although the per-protocol analysis provided useful information to assess the potential effect of the intervention, future larger studies will need to adopt rigorous approaches based on intention-to-treat principles to ensure a more robust and balanced assessment of the effectiveness. Third, it is impossible to guarantee that the participants performed the remote VR-based training session or that another individual replaced them. To date, this drawback is common with most (if not all) home-based training programs. Fourth, in this study, health care workers who volunteered to participate, in addition to having specific demographic characteristics (ie, the majority were female adults with low knowledge of VR), may have been more stressed or receptive to a VR intervention, so the responses may be susceptible to selection bias. The presented results should be replicated in more diverse samples to improve the generalizability of our findings. Fifth, in this study, we used self-reported questionnaires to assess the perceived level of stress and anxiety. Future research should also assess physiological measures related to these conditions (eg, heart rate variability and cortisol levels). In addition, it might be worth investigating the clinical and learning outcomes at follow-up assessments to determine the longer-term effects of remote VR-based training. Finally, in this study, we used only quantitative data, but it could be useful to collect also qualitative feedback to provide deeper insights into the usability and acceptability of the technology, offering valuable perspectives from the participants’ subjective experiences.

### Conclusions

This pilot trial provided clinical outcomes and feasibility results for a remote VR-based intervention for managing stress and anxiety in health care workers. The observed clinical outcomes showed greater improvement in perceived stress as assessed by the PSS-10 and the knowledge of stress and anxiety in the VR group than in the control group. Nevertheless, the findings did not indicate analogous declines in perceived stress, anxiety, and depression levels as gauged by the DASS-21 or in enduring anxiety as evaluated by the STAI-Y1. The home VR training sessions immediately decreased perceived levels of stress and anxiety. The participants found using the VR system and content at home easy to use and without cyber sickness effects. The findings should encourage further research and more extensive studies exploring the potential of VR interventions for delivering psychological support programs in the home, both for caregivers and for other categories of people who need support in managing stress and anxiety.

## Appendix

Multimedia Appendix 1 CONSORT-eHEALTH checklist (V 1.6.1).

## Abbreviations

DASS-21: Depression, Anxiety, and Stress Scale-21 items
HMD: head-mounted display
NPS: Net Promoter Score
PSS-10: Perceived Stress Scale
SDM: Subjective Difficulty Measure
STAI-Y1: State-Trait Anxiety Inventory Form Y-1
STAI-Y2: State-Trait Anxiety Inventory Form Y-2
SUS: System Usability Score
VAS-A: visual analog scale for anxiety
VAS-E: visual analog scale for emotions
VR: virtual reality

## Data Availability

This study’s datasets are available upon request from the authors.
