# Correction: Effects and Acceptability of a 1-Week Home-Based Virtual Reality Training for Supporting the Management of Stress and Anxiety: Randomized Pilot Trial

**DOI:** 10.2196/77791

**Published:** 2025-11-10

**Authors:** Federica Pallavicini, Eleonora Orena, Lisa Arnoldi, Federica Achille, Stefano Stefanini, Maddalena Cassa, Alessandro Pepe, Guido Veronese, Luca Bernardelli, Francesca Sforza, Sara Fascendini, Carlo Alberto Defanti, Marco Gemma, Massimo Clerici, Giuseppe Riva, Fabrizia Mantovani

**Affiliations:** 1 Department of Human Sciences for Education “Riccardo Massa” University of Milano-Bicocca Milan Italy; 2 IRCCS Neurological Institute Carlo Besta Milan Italy; 3 European Biomedical Research Foundation Gazzaniga Italy; 4 Become-Hub Milan Italy; 5 Department of Medicine and Surgery University of Milano-Bicocca Milan Italy; 6 Humane Technology Lab, Department of Psychology Università Cattolica del Sacro Cuore Milan Italy; 7 Applied Technology for Neuro-Psychology Lab IRCCS Istituto Auxologico Italiano Milan Italy

In “Effects and Acceptability of a 1-Week Home-Based Virtual Reality Training for Supporting the Management of Stress and Anxiety: Randomized Pilot Trial” (JMIR Serious Games 2025;13:e50326) the authors noted one error.

In the original article, the affiliation of Giuseppe Riva was incorrectly listed as:

“Applied Technology for Neuro-Psychology Laboratory, IRCCS Italian Auxological Institute, Milan, Italy.”

This has been replaced by:

“Applied Technology for Neuro-Psychology Lab, IRCCS Istituto Auxologico Italiano, Milan, Italy.”

The correction will appear in the online version of the paper on the JMIR Publications website, together with the publication of this correction notice. Because this was made after submission to PubMed, PubMed Central, and other full-text repositories, the corrected article has also been resubmitted to those repositories.

